# The Role of MICA/B Molecules and the NKG2D Receptor in the Interaction Between NK-92 Cells and JEG-3 Cells

**DOI:** 10.3390/ijms26178400

**Published:** 2025-08-29

**Authors:** Elizaveta Tyshchuk, Elizaveta Denisova, Polina Grebenkina, Marina Pereviazkina, Anastasia Stolbovaya, Ilya Smirnov, Olga Shashkova, Irina Gryazeva, Lidiya Terekhina, Dmitry Sokolov

**Affiliations:** 1 Federal State Budgetary Scientific Institution, Research Institute of Obstetrics, Gynecology and Reproductology Named After D.O. Ott, 199034 St. Petersburg, Russia; liza.denisova9898@yandex.ru (E.D.); grebenkinap@gmail.com (P.G.); ujinolga@yandex.ru (O.S.); terehina.l@list.ru (L.T.); 2 Saint-Petersburg Pasteur Institute, 197101 St. Petersburg, Russia; 3 A. Granov Russian Research Center for Radiology and Surgical Technologies, 197758 St. Petersburg, Russia; 4 Department of Immunology, Federal State Budgetary Educational Institution of Higher Education, First St. Petersburg State I. Pavlov Medical University, 197022 St. Petersburg, Russia

**Keywords:** NK cells, trophoblast, MICA/B, NKG2D, trichostatin A

## Abstract

MICA/B molecules (MICs) are stress-induced molecules expressed by infected and tumor cells. Their expression also characterizes trophoblast cells. Cytotoxic lymphocytes, including natural killer (NK) cells, express the NKG2D receptor, aiding them in the recognition and destruction of target cells that present MICs. To evade destruction, target cells employ various defense mechanisms, including the secretion of soluble forms of MICs. Choriocarcinoma JEG-3 cells and NK-92 cells were used to assess the expression of MICs and NKG2D. The cytotoxicity of NK-92 cells against JEG-3 cells in the presence of trichostatin A (TSA), anti-MICA/B antibodies (anti-MICA/B), and recombinant MIC proteins (rMICA/B) was evaluated. JEG-3 cells and NK-92 cells express MICs. Additionally, NK-92 cells exhibit high levels of NKG2D receptor expression. TSA treatment reduced the surface expression of MICs on choriocarcinoma cells, and was also associated with the release of soluble MICB. However, the TSA-induced decrease in MIC expression by choriocarcinoma cells did not protect them from the cytotoxic effects of NK cells. Only the activation of NK cells by IL-12 resulted in a decline in susceptibility of TSA-treated choriocarcinoma cells to NK cell-mediated cytotoxicity. Thus, NK cells activated by IL-12 lose their ability to effectively kill TSA-treated choriocarcinoma cells through the MIC-mediated mechanisms.

## 1. Introduction

MICs are MHC I-like stress-induced molecules, typically expressed at low levels in healthy cells. However, their expression can be upregulated by adverse conditions such as heat shock, viral or bacterial infection, oxidative stress, or malignant transformation [1]. MICs are actively expressed in various types of cancer, including cervical cancer, ovarian cancer, gastric cancer, cholangiocarcinoma, pancreatic cancer, hepatocellular carcinoma, and many others [2,3], as well as in the placenta [4,5]. MICs, along with their related proteins from the ULBP family, serve as ligands for NKG2D, an activating receptor expressed by NK cells, γδ T cells, and CD8+ subsets [6,7,8]. The functional activity and phenotype of NK cells, including NKG2D expression, are regulated by various cytokines, such as IL-12 and IL-15. These cytokines have also been shown to enhance the expression of NKG2D ligands, particularly members of the ULBP family, on peripheral blood NK cells [9]. The binding of NKG2D to MICs results in the transmission of an activating signal to the NK cell [10], triggering their cytotoxic functions [11,12]. To evade immune surveillance, tumors have developed mechanisms that enable the shedding of MICs from the cell surface, resulting in the formation of soluble forms (sMICs). Shedding occurs either through the release of exosomes containing MICs [13,14] or via proteolytic cleavage [15,16]. It has been shown that sMICs, similar to their membrane-bound counterparts, bind to the NKG2D receptor and exert an inhibitory effect on NK cells by downregulating NKG2D expression [14,17]. Thus, sMICs shedding by malignant cells leads to a reduction in the cytotoxic capacity shown by NK cells. In this regard, methods to counteract sMICs are currently being developed. One possible strategy involves the development of antibodies that inhibit the sMICs shedding or their binding with NKG2D [18]. The use of antibodies targeting MICs presents a promising strategy for tumor treatment. In addition, antibodies to MICs could be employed in the treatment of reproductive pathologies associated with insufficient NK cell activity. However, there are currently insufficient data on the role of MICs in intercellular interactions between NK cells and choriocarcinoma cells. Existing research on this topic hints that certain substances can enhance the MIC expression. Specifically, histone deacetylase inhibitors (HDACis), such as TSA [19], as well as some cytokines and cellular stress inducers, have been shown to upregulate MIC expression [19,20]. The effects of these inducers on the MIC production by NK cells and choriocarcinoma cells remain unexplored.

Thus, the aim of the present study was to investigate the role of MICs in the interaction between NK cells and choriocarcinoma cells. In our study, we used rMICA/B and anti-MICA/B to evaluate their effect on the interaction between NK cells and their target cells, JEG-3 and K-562. The rMICA/B addition was intended to simulate an excess of sMICs in the cell culture medium, whereas the introduction of anti-MICA/B aimed to isolate sMIC forms.

## 2. Results

### 2.1. TSA as a Potential Cause for Decline in MIC-Expressing Cells

**Figure 1:**
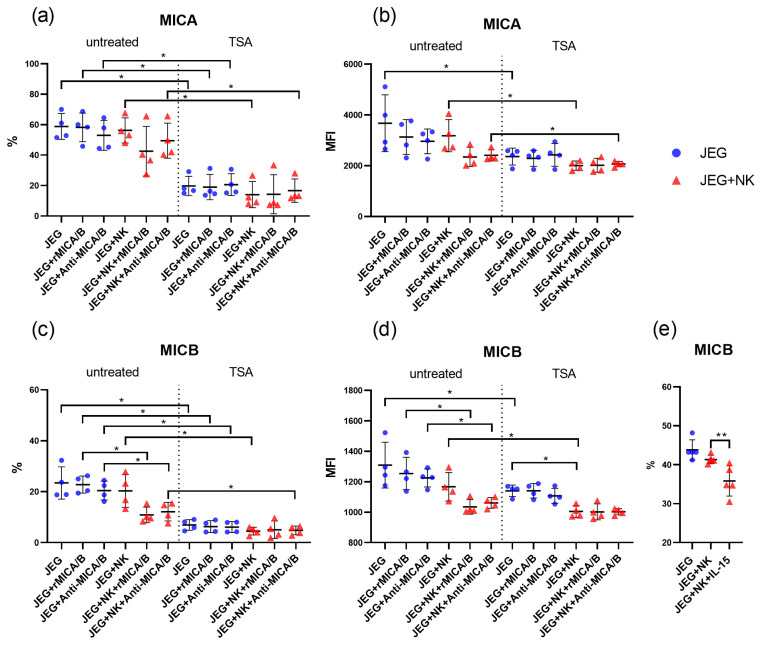
Phenotype of JEG-3 cells under mono- and co-culture conditions. The figure shows the percentage (%) of JEG-3 cells expressing MICA (**a**) and MICB (**c**,**e**) proteins, as well as the intensity of expression (MFI) of MICA (**b**) and MICB (**d**). Some cells were treated with TSA (**a**–**d**), rMICA/B, and anti-MICA/B. Some NK cells were activated with IL-15 (**e**). MFI—Median Fluorescence Intensity; untreated—TSA-untreated cells; TSA—TSA-treated cells; JEG—JEG-3 cells; NK—NK-92 cells. Significant differences: *—*p* < 0.05; **—<0.01. There were four biological replicates with one technical replicate in each experiment. The full version of the figure demonstrating the changes in the JEG-3 cell phenotype is presented in Supplementary Figure S2.

JEG-3 cells express MICA and MICB molecules (Figure 1, Supplementary Figure S1). JEG-3 cells’ treatment with rMICA/B or anti-MICA/B did not induce changes in the expression of MICA and MICB (Figure 1a–d).

JEG-3 cells’ treatment with TSA showed a decline in MIC-expressing cells versus untreated controls, with or without rMICA/B or anti-MICA/B (Figure 1a,c). However, the intensity of MIC expression for JEG-3 cells after TSA treatment was lower than for TSA-untreated cells. This difference was observed only for cells cultured in the absence of rMICA/B or anti-MICA/B (Figure 1b,d).

### 2.2. Downregulation of MICB in JEG-3 Cells by NK Cells in the Presence of rMICA/B or Anti-MICA/B

JEG-3 cells did not alter the expression of MICA and MICB proteins in the presence of NK-92 cells compared with monoculture conditions. However, the addition of rMICA/B or anti-MICA/B to the co-culture led to a reduction in MICB expression by JEG-3 cells compared with monoculture levels (Figure 1c,d). In contrast, the MICA expression by JEG-3 cells remained unchanged under all experimental conditions (Figure 1a,b). IL-15-treated NK-92 cells reduced MICB-expressing JEG-3 cells compared with untreated NK-92 co-cultures (Figure 1e).

JEG-3 cells co-cultured with NK cells and TSA showed lower MICB expression intensity than TSA-treated monocultures (Figure 1d). Treatment of cell co-culture with TSA reduced the number of JEG-3 cells expressing MICs compared with untreated co-culture. A similar reduction was observed for the co-culture treated with anti-MICA/B. However, when the co-culture was treated with rMICA/B, the difference in the number of cells expressing MICs disappeared (Figure 1a,c). This effect is probably caused by the ability of rMICA/B to downregulate the MICB expression by JEG-3 cells when co-cultured with NK-92 cells (Figure 1c,d). Additionally, TSA-treated co-culture decreased in MIC expression intensity by JEG-3 cells versus negative control (Figure 1b,d).

### 2.3. The Effect of TSA on MICA Expression and NK Cell Phenotype Modulation by IL-12, rMICA/B, and Anti-MICA/B

**Figure 2:**
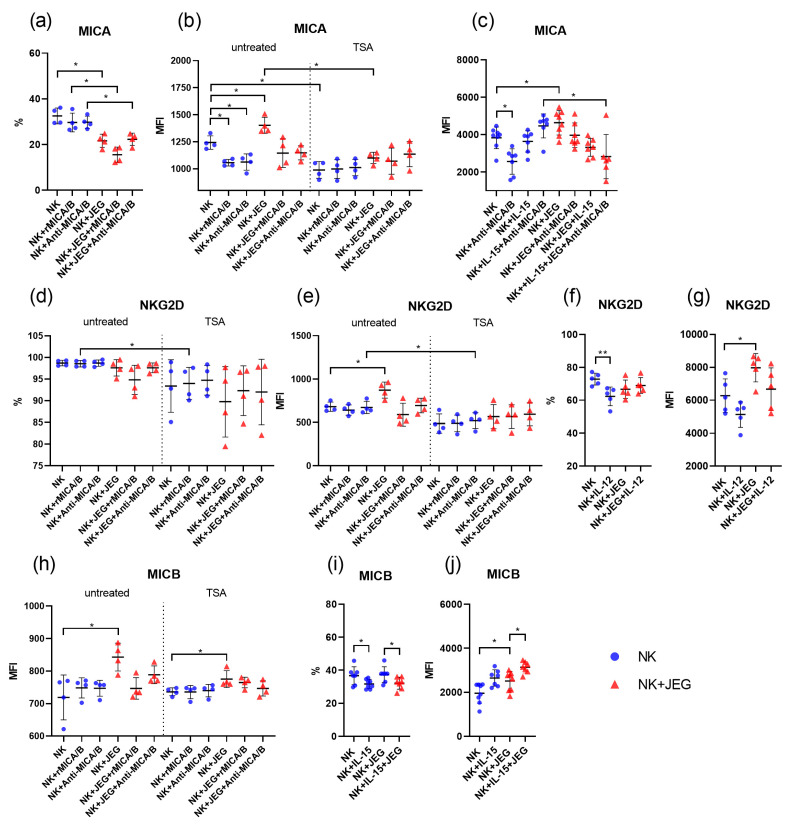
Phenotype of NK-92 cells under mono- and co-culture conditions. The figure shows the percentage (%) of NK-92 cells expressing MICA (**a**), NKG2D (**d**,**f**), and MICB (**i**) proteins, as well as the intensity of expression (MFI) of MICA (**b**,**c**), NKG2D (**e**,**g**), and MICB (**h**,**j**). Some cells were treated with TSA (**b**,**d**,**e**,**h**), rMICA/B, and Anti-MICA/B. Some NK cells were activated with IL-12 (**f**,**g**) or IL-15 (**i**,**j**). MFI—Median Fluorescence Intensity; untreated—TSA-untreated cells; TSA—TSA-treated cells; JEG—JEG-3 cells; NK—NK-92 cells. Significant differences: *—*p* < 0.05; **—<0.01. There were four biological replicates with one technical replicate in each experiment. The full version of the figure demonstrating the changes in the NK-92 cell phenotype is presented in Supplementary Figure S3.

The addition of rMICA/B or anti-MICA/B to NK-92 cells led to a decrease in the MICA expression intensity but did not affect the number of MICA-expressing cells versus untreated controls (Figure 2a,b). The addition of rMICA/B or anti-MICA/B to NK-92 cells did not cause changes in MICB and NKG2D expression compared with untreated cells (Supplementary Figure S3(a1,a2,c1,c2)). Treatment of NK-92 cells with IL-12 cells resulted in a decline in the number of cells expressing the NKG2D receptor, though the intensity of its expression remained unchanged relative to IL-12-untreated cells (Figure 2f,g). Treatment of NK-92 cells with IL-12 did not affect the MICA and MICB expression by NK-92 cells (Supplementary Figure S3(e1,e2,f1,f2)). After treatment with IL-15, the number of NK-92 cells expressing MICB lowered, although the intensity of MICB expression remained unchanged compared with untreated cells (Figure 2i,j). Additionally, IL-15 treatment did not influence the expression of MICA or NKG2D by NK-92 cells (Supplementary Figure S3(d1,d2,e1,e2)).

Treatment of NK-92 cells with TSA resulted in a decrease in MICA expression compared with untreated cells (Figure 2b). However, no changes were observed in the expression of MICB or NKG2D (Supplementary Figure S3(a1,a2,c1,c2)). The addition of rMICA/B to TSA-treated NK-92 cells caused a decrease in NKG2D expression relative to untreated cells, while the anti-MICA/B addition resulted in a reduction in the NKG2D expression intensity (Figure 2d,e).

### 2.4. JEG-3 Cells’ Effects on NK Phenotype

When co-cultured with JEG-3 cells, the number of NK-92 cells expressing MICA was lower compared with monoculture conditions. This effect was also observed in the presence of rMICA/B or anti-MICA/B (Figure 2a). At the same time, under co-culture conditions with JEG-3, the intensities of MIC and NKG2D expression by NK cells were higher than under monoculture conditions (Figure 2b,e,h). However, the addition of rMICA/B or anti-MICA/B reversed this effect: the intensity of MIC and NKG2D expression decreased to levels comparable to those observed in monoculture (Figure 2b,e,h). This suggests that rMICA/B and anti-MICA/B influence the interaction between NK-92 and JEG-3 cells when co-cultured. Treatment with IL-12 did not affect the MIC and NKG2D expression by NK cells in the presence of JEG-3 cells (Supplementary Figure S3(d1,d2,e1,e2,f1,f2)). Treatment of NK cells with IL-15 followed by co-culturing with JEG-3 cells caused a decline in NK cells expressing MICB compared with the untreated control. However, the intensity of MICB expression increased (Figure 2i,j). Treatment of NK cells with IL-15 and subsequent co-culturing with JEG-3 cells and anti-MICA/B resulted in a decrease in the MICA expression intensity by NK cells compared with IL-15-treated NK cells monocultured with anti-MICA/B (Figure 2c).

TSA treatment reduced MICA expression in NK-92 cells co-cultured with JEG-3 cells compared with untreated controls. This effect was not observed when rMICA/B or anti-MICA/B was added to the cell co-culture, which is probably due to the ability of rMICA/B and anti-MICA/B to reduce the intensity of MICA expression by NK cells (Figure 2b). Treatment of NK-92 and JEG-3 co-culture with TSA caused an increase in the intensity of MICB expression on NK cells compared with the monoculture conditions. The addition of rMICA/B or anti-MICA/B to the co-culture reversed this effect, reducing the intensity of MICB expression by NK cells to the level observed in monoculture (Figure 2h).

### 2.5. sMICB Secretion by JEG-3 Cells Stimulated with TSA

Tumor and placental cells are able to shed MICs, which allows them to attenuate the cytotoxic activity of NK cells. In this regard, we evaluated the ability of JEG-3 trophoblast cells to release sMICs, including after treatment with a TSA inducer.

**Figure 3:**
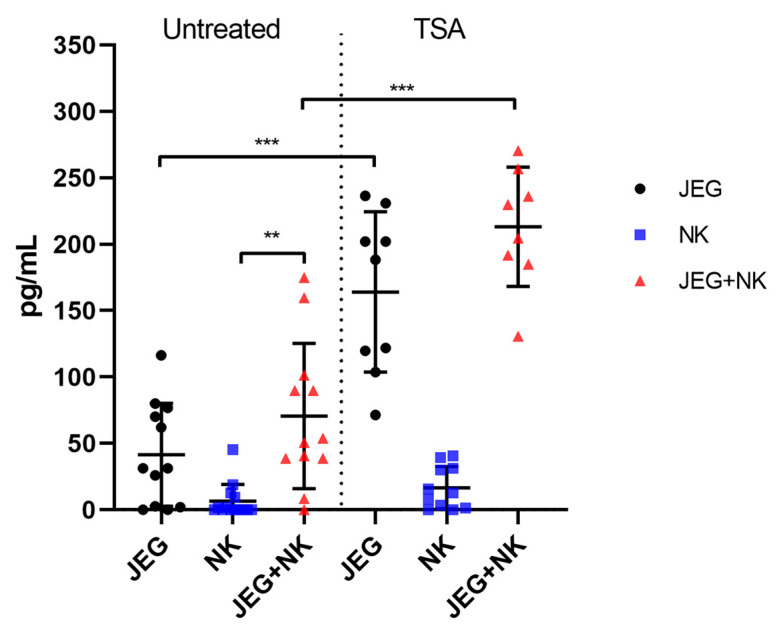
The sMICB concentration (pg/mL) in the medium after the mono- and co-culturing of NK-92 and JEG-3 cells. Untreated—TSA-untreated cells; TSA—TSA-treated cells. Supernatants were obtained from three independent experiments with at least 3 technical replicates. Significant differences: **—*p* < 0.01; ***—<0.001.

The ELISA showed that JEG-3 cells, but not NK-92 cells, produce the soluble form of the MICB protein (sMICB) (Figure 3). Co-cultivation of JEG-3 cells with NK-92 cells did not affect the production of sMICB by JEG-3 cells. The addition of TSA to the cells caused an increase in sMICB levels in the culture medium obtained from JEG-3 cells and co-cultured cells (Figure 3). The soluble form of the MICA protein was not detected during the assay.

### 2.6. TSA Enhanced the Death Rates of K-562 and JEG-3 Cells, but This Effect Was Not Observed in the Presence of NK Cells or PBMCs

Binding of the NK cell receptor NKG2D to MICs expressed by target cells triggers cytotoxic activity in NK cells. We investigated how the cytotoxicity of NK-92 cells towards JEG-3 trophoblast cells is modulated in the presence of rMICA/B or anti-MICA/B. Additionally, JEG-3 cells were treated with TSA, while NK cells were activated using the cytokines IL-12 and IL-15. To further validate the findings, cytotoxic activity was also assessed using K-562 cells, which are widely used as target cells in cytotoxicity studies [21,22,23]. To confirm the results obtained with the NK-92 cell line, additional experiments were performed using peripheral blood mononuclear cells (PBMCs) as effector cells. In all the experiments, the death rates of target cells were significantly higher following incubation with effector cells.

**Figure 4:**
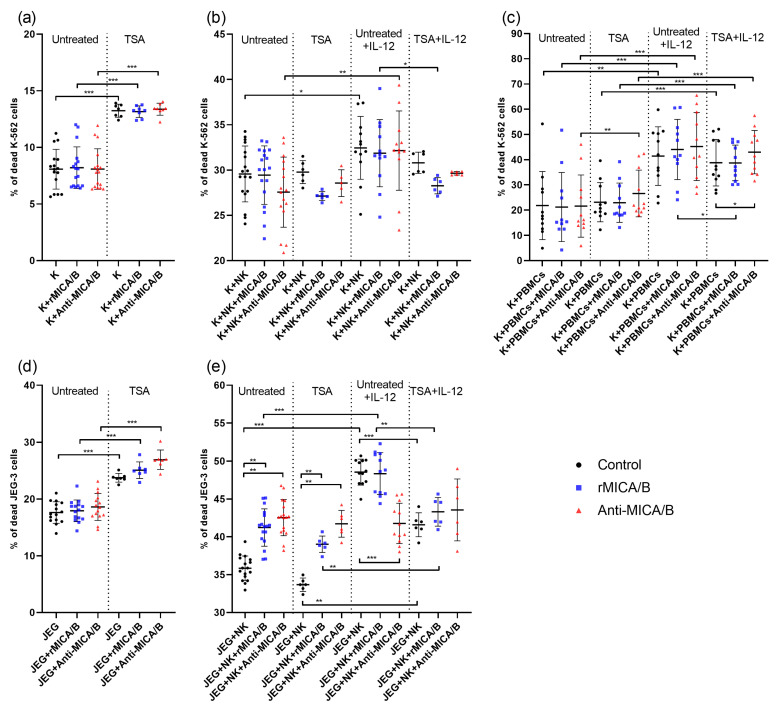
Cytotoxicity of NK-92 cells (**a**,**b**,**d**,**e**) and PBMCs (**c**) toward target cells. The percentage of dead K-562 (**a**) and JEG-3 (**d**) cells, untreated or pre-incubated with TSA in the presence of rMICA/B or anti-MICA/B. The percentage of dead K-562 (**b**) and JEG-3 (**e**) cells after co-culturing with untreated or IL-12-activated NK-92 cells. The percentage of dead K-562 (**c**) after co-culturing with untreated or IL-12-activated PBMCs. The death level of target cells under monoculture conditions was significantly lower in all cases than after co-culturing with NK cells. For (**a**,**b**,**d**,**e**), each series included three experiments with at least two technical repetitions. For (**c**), PBMCs from 12 donors were used. Untreated—TSA-untreated targets; TSA—TSA-treated targets; Untreated + IL-12—TSA-untreated targets with IL-12-activated NK cells or PBMCs; TSA+IL-12—TSA-treated targets with IL-12-activated NK cells or PBMCs; K—K-562 cells; JEG—JEG-3 cells; NK—NK-92 cells; PBMCs—peripheral blood mononuclear cells. Significant differences: *—*p* < 0.05; **—<0.01; ***—<0.001.

Treatment of target cells with TSA led to an increase in their death rates compared with untreated cells (Figure 4a,d). However, under co-culture conditions with NK cells, the death rates of TSA-treated target cells did not differ from those of TSA-untreated cells (Figure 4b,e). Similar results were observed when K-562 cells were co-cultured with PBMCs (Figure 4c). Thus, while TSA treatment exerted a toxic effect on target cells, it also triggered mechanisms that enabled the cells to resist the cytotoxic activity of NK cells and PBMCs.

### 2.7. Enhanced NK Cell Cytotoxicity Against JEG-3 Cells with rMICA/B or Anti-MICA/B

The addition of rMICA/B or Anti-MICA/B to target cells did not affect their death rates, indicating the absence of toxic effect of rMICA/B and anti-MICA/B on cells (Figure 4a,d). Furthermore, the addition of rMICA/B or anti-MICA/B did not influence the mortality of K-562 cells in the presence of NK cells, including under TSA treatment conditions (Figure 4b). Under conditions of co-culture with PBMCs and anti-MICA/B, K-562 cells treated with TSA showed a higher death rate than TSA-untreated cells (Figure 4c). The addition of rMICA/B or anti-MICA/B to the co-culture of JEG-3 and NK cells increased the mortality of JEG -3 cells compared with a co-culture control. This effect was also observed for TSA-treated JEG-3 cells (Figure 4e).

### 2.8. IL-12-Activated NK/PBMC Cytotoxicity and JEG-3 Survival in the Presence of Anti-MICA/B

In this study, NK cells and PBMCs were activated using IL-12 and IL-15 to enhance their cytotoxic activity. Activation with IL-15 did not lead to significant results. Activation of NK cells with IL-12 enhanced their cytotoxicity towards K-562 cells, including in the presence of anti-MICA/B (Figure 4b). Activation of PBMCs with IL-12 also increased their cytotoxicity towards K-562 cells. Treatment of K-562 cells with TSA and addition of rMICA/B or anti-MICA/B did not reverse this effect (Figure 4c). The rMICA/B or anti-MICA/B addition to the co-culture of IL-12-activated NK cells and K-562 cells did not alter the K-562 cells’ death rate. However, the mortality of K-562 cells co-cultured with IL-12-activated NK cells and rMICA/B was the same as that observed in the presence of non-activated NK cells and rMICA/B (Figure 4b). As for JEG-3 cells, the anti-MICA/B addition to the co-culture of JEG-3 and IL-12-activated NK cells resulted in a significant decrease in the death rate, compared with that observed without anti-MICA/B (Figure 4e). The mortality of JEG-3 cells observed in this case returned to the level shown during incubation with non-activated NK cells (Figure 4e).

### 2.9. TSA-Mediated Protection of Target Cells from IL-12-Activated NK/PBMC Cytotoxicity in the Presence of rMICA/B

Activation with IL-12 did not affect NK cell-mediated cytotoxicity towards TSA-treated K-562 cells: the viability of treated and untreated target cells did not differ, including in the presence of rMICA/B or anti-MICA/B. (Figure 4b). However, only in the presence of IL-12-activated NK cells, the death rate of K-562 cells in the presence of rMICA/B was significantly lower for TSA-treated cells than for TSA-untreated cells (Figure 4b). The same effect was observed for IL-12-activated PBMCs (Figure 4c). In addition, an increase in the death rate of K-562 cells was observed under conditions of TSA treatment and co-culture with IL-12-activated PBMCs and anti-MICA/B (Figure 4c). Activation of NK cells by IL-12 also enhanced their cytotoxicity against JEG-3 cells treated with TSA, including in the presence of rMICA/B (Figure 4e). Moreover, in the case of JEG-3 cells, a decrease in mortality was also found after the TSA treatment. Thus, TSA-treated cells, including with rMICA/B, showed a lower death rate in the presence of IL-12-activated NK cells compared with TSA-untreated JEG-3 cells (Figure 4e). Therefore, the supposed ability of TSA to reduce the target death in the presence of NK cells was confirmed after their activation by IL-12 and rMICA/B introduction.

## 3. Discussion

MICA/B molecules indicate cellular stress and are typically expressed in many types of tumors [2]. This study showed that JEG-3 choriocarcinoma cells express MICs and also secrete sMICB. MICs’ mRNA is found in placental samples [4,5], and sMICs are present in amniotic fluid [24]. Thus, the expression of MICs by JEG-3 cells is determined not only by their tumor origin, but also by their belonging to trophoblast cells. We have also evaluated the MICs’ expression by NK cells of the NK-92 cell line. No data on the expression of MICs by NK-92 cells or NK cells derived from peripheral blood have been found in the literature. However, there is evidence that CD4+ T cells and decidual antigen-presenting cells express MICA and other NKG2D ligands [25,26]. The expression of MICs by lymphocytes may indicate the existence of a mechanism for the self-regulation of immune activity by NKG2D-expressing lymphocytes in pathological conditions [27,28]. NK-92 cells also showed a high level of expression of the MICs’ receptor, NKG2D. Based on these data, we believe that the cell lines used in this study are suitable for assessing the role of MICs in intercellular communication between choriocarcinoma cells and natural killer cells.

In this study, we used TSA to induce MIC expression. rMICA/B and anti-MICA/B were also used to simulate excess or deficiency of MICs in the cellular system, respectively. As with other HDACis, TSA was expected to upregulate MIC expression in choriocarcinoma cells. However, no such effect was observed in JEG-3 cells. We showed that TSA treatment resulted in the release of a membrane-bound MICB and the formation of a soluble one. Furthermore, MICA expression by choriocarcinoma cells was reduced following TSA treatment, although no sMICA was detected. The addition of rMICA/B or anti-MICA/B to the cells did not lead to changes in the number of cells expressing MICs, indicating the absence of competition between the reagents used and the fluorescent antibodies applied during cytometry. In this study, we used the *008 allelic variant of MICA protein and the *005 allelic variant of MICB protein, as well as three clones of antibodies for each protein [29]. These protein variants are among the most prevalent in human populations [14,30,31]. Moreover, it was established that the antibodies we used are capable of binding different allelic variants of MICs [29]. The use of antibodies results in various effects depending on the epitope of the molecule they bind to. For example, the authors of one study described the ability of antibodies they developed to bind to the α-3 domain of MICs. This causes a significant increase in the expression of surface MICs and disrupts the synthesis of sMICs [18]. One of the three antibodies to MICB used in this work also binds to the α-3 domain, but treatment with it does not lead to disruption of MICB shedding. It has also been established that the use of antibodies binding to membrane forms of MICs activates Fc-dependent cytotoxicity mechanisms in NK cells [32]. NK-92 cells do not express the Fc receptor (CD16) [33]; however, we analyzed the cytotoxic activity of PBMCs. NK cells and macrophages within the PBMCs could potentially be activated in response to targets treated with anti-MICA/B. Indeed, the antibodies we used contributed to an increase in target cell death rates, but only under conditions where the targets were treated with TSA and PBMCs were activated by IL-12.

According to the literature, sMICs have an inhibitory effect on NK cells and CD8+ T cells, reducing their expression of NKG2D and inhibiting their cytotoxic activity [14,17]. It is also hypothesized that the binding of sMICs to NKG2D may lead to the internalization of this receptor [32]. We have not detected a decrease in NKG2D receptor expression by NK-92 NK cells after 24 h incubation in the presence of rMICA/B. Moreover, co-culture with rMICA/B and JEG-3 choriocarcinoma cells, which release sMICs into the medium, also did not lead to a decrease in NKG2D receptor expression by NK cells. On the contrary, co-culture with JEG-3 cells caused an increase in the intensity of NKG2D expression by NK cells. In one of the studies mentioned, it was specified that NK cells were treated with exosomes containing MICA*008 [14], which may explain the differences in the results obtained. In addition, in one study, the addition of IL-2 to NK cells restored their expression of NKG2D to its previous level [34]. Perhaps due to the use of IL-2 as a growth factor for NK-92 cells, we did not find a decrease in their NKG2D expression.

The signal from NKG2D is critical for the activation of NK cells, as it overrides the signals transmitted by their inhibitory receptors [8]. Thus, we can assume that the binding of NKG2D to MICs would inevitably lead to the death of target cells. However, this does not occur, as confirmed by the obtained results: only 30–40% of the target cell population died after a 4 h incubation with NK cells, while NKG2D expression was observed in more than 70% of the NK-92 cell population. In one study focused on MICs, the authors concluded that sMICs and membrane-bound MICs exert different effects on NK cells [35]. Specifically, sMICs suppress the cytotoxic functions of NK cells but promote the synthesis of various inflammatory and protumorigenic factors (RELB, CCL1, CCL3, NFKB2, CCL4, IL-10), whereas membrane-bound MICs stimulate cellular cytotoxicity. We investigated how the death rate of choriocarcinoma cells changed during co-culture with NK cells. Since TSA treatment or the addition of rMICA/B to cells leads to an increase in sMIC levels in the medium, we hypothesized that the cytotoxicity of NK cells would decrease. Conversely, the addition of anti-MICA/B was expected to reduce sMIC levels, although it does not exclude the possibility of sMICs’ interaction with NKG2D. Contrary to our expectations, we found that treatment of target cells with TSA had no effect on their death rates in the presence of NK cells. The addition of rMICA/B or anti-MICA/B to the co-culture of NK cells and choriocarcinoma cells, but not K-562 cells, increased choriocarcinoma cell death in both cases, contradicting our initial assumptions. This suggests that, when interactions based on MIC recognition are disrupted, alternative modes of communication may come into play. For instance, unlike K-562 cells, trophoblast cells express non-classical HLA molecules and other proteins recognized by activating receptors on NK cells [36,37].

In this study, we treated NK cells with IL-12 and IL-15 to enhance their cytotoxic properties. Previous studies have reported that treatment with IL-12 increases the transcription levels and surface expression of NKG2D in NK cells [38]. However, contrary to these findings, we observed that activation with IL-12 reduced the expression of NKG2D on the cells. Nevertheless, NK cells activated by IL-12 were more efficient at destroying target cells, likely due to the activation of alternative killing mechanisms involving other NK cell receptors.

## 4. Materials and Methods

### 4.1. Cell Lines

For this study, we used the K-562 (lymphoblast cells), JEG-3 (choriocarcinoma, extravillous trophoblast cells), and NK-92 (natural killer cells) cell lines (American Type Culture Collection (ATCC, Manassas, VA, USA) [33,39,40]. K-562 cells were cultured in RPMI medium (Biolot, Saint-Petersburg, Russia, 1.3.4.) supplemented with 10% FBS (Biolot, Saint-Petersburg, Russia, 1.1.8.1.), 100 μg/mL streptomycin and 100 IU/mL penicillin (Biolot, Saint-Petersburg, Russia, 1.3.18.), and 2 mM L-glutamine (Sigma, St. Louis, MO, USA, G8540). Subculturing was performed every 2 days. JEG-3 cells were cultured in DMEM medium (Biolot, Saint-Petersburg, Russia, 1.3.5.) supplemented with 10% FBS (Biolot, Saint-Petersburg, Russia, 1.1.8.1.), 100 μg/mL streptomycin and 100 IU/mL penicillin (Biolot, Saint-Petersburg, Russia, 1.3.18.), 2 mM L-glutamine (Sigma, St. Louis, MO, USA, G8540), 1% non-essential amino acids (Biolot, Saint-Petersburg, Russia, 1.3.23.), and 1 mM sodium pyruvate (Sigma, St. Louis, MO, USA, P2256). Subculturing was performed once every 3–4 days. Monolayer detachment was achieved by exposure to a 1:1 solution of trypsin (Biolot, Saint-Petersburg, Russia, 1.2.5.5.) and versene (Biolot, Saint-Petersburg, Russia, 1.2.3.2.). NK-92 cells were cultured in α-MEM medium (Biolot, Saint-Petersburg, Russia, 1.3.9.4.) supplemented with 10% FBS (Biolot, Saint-Petersburg, Russia, 1.1.8.1.), 10% horse serum (Biolot, Saint-Petersburg, Russia, 1.1.5.8.), 0.2 mM myo-inositol (Sigma, St. Louis, MO, USA, 57570), 0.02 mM folic acid (Sigma, St. Louis, MO, USA, F8758), 2 mM L-glutamine (Sigma, St. Louis, MO, USA, G8540), 100 μg/mL streptomycin and 100 IU/mL penicillin (Biolot, Saint-Petersburg, Russia, 1.3.18.), 10 mM HEPES buffer (Biolot, Saint-Petersburg, Russia, 1.2.6.1.), 0.1 mM 2-mercaptoethanol (Sigma, St. Louis, MO, USA, M3148), and 500 IU/mL recombinant IL-2 (“Roncoleukin”; LLC “Biotech”, Saint-Petersburg, Russia). Subculturing was performed every 2 days. Cells were cultured, and all experiments were carried out in a humidified incubator at 37 °C with 5% CO_2_. Cell viability was assessed in all experiments using trypan blue dye; viability was at least 95% in all the experiments.

### 4.2. Inductors

Recombinant IL-12 (10 ng/mL, Cat. SRP3073, Sigma, St. Louis, MO, USA) and recombinant IL-15 (10 ng/mL, Cat. SRP3077, Sigma, St. Louis, MO, USA) were used as activators for NK cells. TSA (1 μg/mL, Cat. T8552, Sigma, St. Louis, MO, USA); Supplementary Figure S4) was employed as an inductor of MIC expression [19,41]. Recombinant MICA*008 and MICB*005 were used in combination with each protein at a concentration of 100 ng/mL. Monoclonal antibodies targeting MICA (clone 1E2, 2G1, and 4A11) and MICB (clone 1B8, 2A8, and 4D11) were also used in combination, with each antibody at a concentration of 10 μg/mL. The recombinant proteins and antibodies were produced following the protocol described in [29]. All concentrations were chosen based on information from the literature.

### 4.3. Assessment of the Phenotype of JEG-3 and NK-92 Cells Following Mono- and Co-Culture in the Presence of Inducers

**Figure 5:**
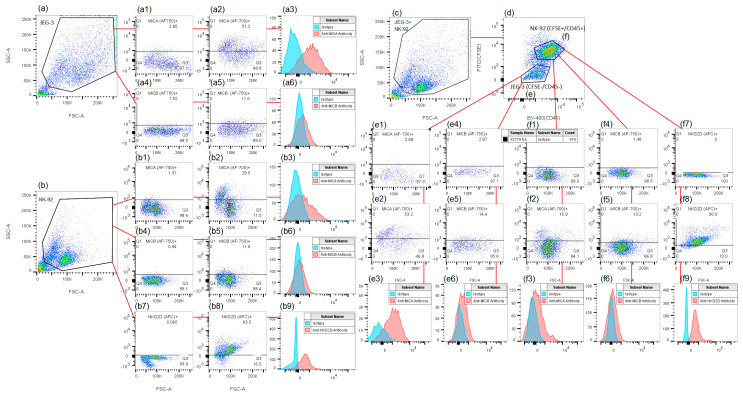
A gating strategy used for the estimation of NK-92 and JEG-3 cells’ phenotype after co-culturing. (**a**–**c**)—two-dimensional histograms showing the JEG-3 (**a**), NK-92 (**b**), and JEG-3+NK-92 (**c**) cell distributions in FSC/SSC coordinates. (**a1**–**a6**)—histograms showing the MICA (AF-700) (**a1**–**a3**) and MICB (AF-750) (**a4**–**a6**) expression by JEG-3 cells in monoculture conditions. (**b1**–**b9**)—histograms showing the MICA (AF-700) (**b1**–**b3**), MICB (AF-750) (**b4**–**b6**), and NKG2D (APC) (**b7**–**b9**) expression by NK-92 cells in monoculture conditions. (**d**)—a two-dimensional histogram showing the distribution of NK-92 cells treated with CFSE and anti-CD45 antibodies, as well as JEG-3 cells treated with anti-CD45 antibodies in CFSE (FITC)/CD45 (BV480) coordinates. (**e1**–**e6**)—histograms showing the MICA (AF-700) (**e1**–**e3**) and MICB (AF-750) (**e4**–**e6**) expression by JEG-3 cells after co-culturing with NK-92 cells. (**f1**–**f9**)—histograms showing the MICA (AF-700) (**f1**–**f3**), MICB (AF-750) (**f4**–**f6**), and NKG2D (APC) (**f7**–**f9**) expression by NK-92 cells after co-culturing with JEG-3 cells. (**a1**,**b1**,**e1**,**f1**)—isotype controls for MICA; (**a4**,**b4**,**e4**,**f4**)—isotype controls for MICB; (**b7**,**f7**)—isotype controls for NKG2D; (**a2**,**b2**,**e2**,**f2**)—anti-MICA antibodies; (**a5**,**b5**,**e5,f5**)—anti-MICB antibodies; (**b8**,**f8**)—anti-NKG2D antibodies; (**a3**,**a6**,**b3**,**b6**,**b9**,**e3**,**e6**,**f3**,**f6**,**f9**)—the difference in the intensity of protein expression following treatment with isotypic controls versus specific antibodies.

JEG-3 and NK-92 cells were cultured separately in 24-well flat-bottomed plates (Corning, Glendale, AZ, USA) at a concentration of 0.12 × 10^6^ cells per well in 1 mL of complete medium. In some wells containing NK-92 cells, IL-12 or IL-15 was added as an activator. After 22 h, NK-92 cells were stained with CFSE according to the manufacturer’s instructions (Sigma, St. Louis, MO, USA). Subsequently, some wells containing JEG-3 cells were treated with TSA, rMICA/B, or anti-MICA/B. After 20 min of incubation, NK-92 cells were added to some of the wells with JEG-3 cells to establish co-culture conditions. Intact JEG-3 cells and CFSE-stained NK-92 cells were used as controls. Following 22 h of incubation, the cells were removed from the plates without the use of a trypsin-versene solution. The cells were then treated with an Fc-block solution (Miltenyi Biotec, Madrid, Spain) and fluorescent monoclonal antibodies, following the manufacturer’s instructions. Isotypic antibodies were used as controls for non-specific binding. The phenotype of the cells was analyzed using a FacsCantoII flow cytometer (BD, Franklin Lakes, NJ, USA). There were four biological replicates with one technical replicate in each experiment. Two independent series of experiments were conducted. In the first, the phenotype of TSA-treated JEG-3 and NK-92 cells was analyzed. In the second, changes after co-culturing JEG-3 cells with IL12/IL-15-activated NK-92 cells were measured. Different antibody panels were used in each series (Table 1). The gating strategy is shown in Figure 5. For a graphical representation of this method, please refer to Supplementary Figure S5.

### 4.4. Assessment of the Cytotoxic Activity of NK-92 Cells Against JEG-3 and K-562 Cells and PBMCs Against K-562 Cells

**Figure 6:**
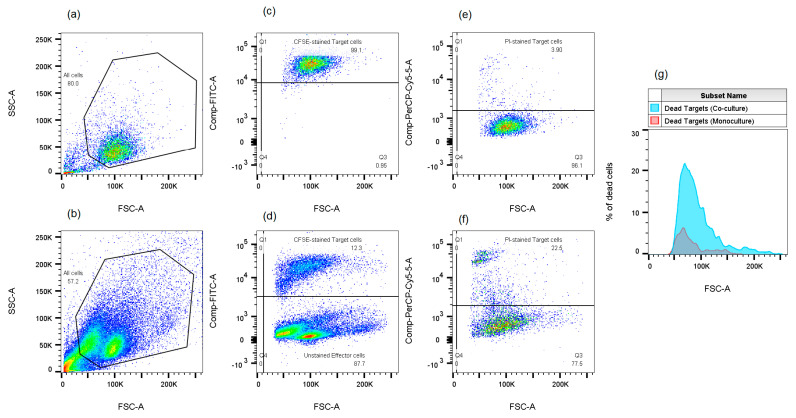
Evaluation of cytotoxic activity of effector cells (NK-92 cells/PBMCs) against target cells (JEG-3/K-562 cells). (**a**,**b**)—two-dimensional histograms showing the distribution of K-562 target cells under monoculture (**a**) and co-culture conditions with NK-92 cells (**b**) in FSC/SSC coordinates. (**c**,**d**)—two-dimensional histograms showing the distribution of CFSE-treated K-562 cells under monoculture (**c**) and co-culture conditions with untreated NK-92 cells (**d**) in CFSE (FITC)/FSC coordinates. (**e**,**f**)—two-dimensional histograms showing the distribution of CFSE-treated K-562 cells stained with propidium iodide (PI) under monoculture (**e**) and co-culture conditions with NK-92 cells (**f**) in PI (PerCP-Cy5-5)/FSC coordinates. (**g**)—a histogram showing the difference in the number of dead K-562 cells under monoculture versus co-culture conditions with NK-92 cells.

The test was conducted using a standard protocol [42]. Twenty-four hours prior to the experiment, NK-92 (effector cells), as well as JEG-3 and K-562 cells (target cells), were seeded into new flasks. PBMCs were isolated from whole blood using density gradient centrifugation (400× *g* for 40 min) using Ficoll solution (density 1.077/mL, Biolot, Saint-Petersburg, Russia) following the protocol recommended by Sigma-Aldrich. The isolated PBMCs were then placed in a 24-well plate. IL-12 was added to some flasks and wells containing NK cells or PBMCs. TSA was added to some flasks containing target cells. The next day, the target cells were stained with CFSE solution following the manufacturer’s instructions (Sigma, St. Louis, MO, USA) and placed into a 96-well round-bottom plate (BD, USA). For JEG-3 cells, a trypsin and versene solution was used to remove the cells. Some target cells were treated with rMICA/B or anti-MICA/B for 20 min. After that, NK-92 cells or PBMCs were added to the wells containing target cells at an effector-to-target ratio of 10:1. The plate was centrifuged for 5 min at 100× *g* to facilitate cell–cell contact. After 4 h of incubation, the cells were stained with propidium iodide solution according to the manufacturer’s instructions (Sigma, St. Louis, MO, USA). The relative number of dead target cells was assessed using a FacsCanto II flow cytometer (BD, Franklin Lakes, NJ, USA). The research was conducted in accordance with the Code of Ethics of the World Medical Association (i.e., the Helsinki Declaration) and was approved by the local ethical committee of the Federal State Budgetary Scientific Institution “Research Institute of Obstetrics, Gynecology, and Reproductology named after D.O. Ott” (Protocol No. 118, 9 June 2022). For NK-92 cells, three experiments with at least two technical replicates each were performed; for PBMCs, blood from 12 donors was used. The gating strategy is shown in Figure 6. For a graphical representation of this method, please refer to Supplementary Figure S6.

### 4.5. Enzyme-Linked Immunosorbent Assay

JEG-3 cells were cultured in 24-well flat-bottomed plates at a concentration of 0.1 × 10^6^ cells per well in 0.8 mL of complete medium. After 22 h of incubation, 0.15 × 10^6^ NK-92 cells in 0.2 mL of complete medium were added to the wells with JEG-3 cells. After that, TSA was added to some wells with either mono- or co-cultures. Intact NK-92 and JEG-3 cells were used as controls and were incubated in 1 mL of medium. After 22 h, the plates were centrifuged at 100× *g* for 5 min, and the supernatants were collected and stored at −20 °C. sMIC levels in the samples were quantified by two-site ELISA. A total of three biological replicates were performed, with at least three technical replicates per experiment. For a graphical representation of this method, please refer to Supplementary Figure S7.

### 4.6. Quantitative RT-qPCR

mRNA levels in JEG-3 were assessed by quantitative reverse transcription PCR (RT-qPCR). Total RNA was isolated using the standard phenol–chloroform extraction method. An amount of 2 μg of total RNA was reverse-transcribed into complementary DNA using a commercial kit (Evrogen, SK022S, Moscow, Russia). RT-qPCR was then performed on a DT-96 amplifier (DNA Technology, Russia) using SYBR green PCR mix (Evrogen, PK147S, Russia) under the following program: cycle 1 (1 cycle): 95°—3 min; cycle 2 (35 cycles): 95°—10 s, 60°—10 s, 72°—20 s; cycle 3 (melting curve, 140 steps): 90°—15 s; cycle 4 (hold)—4°. The amplification cycles (Ct values) for MICA, MICB, and the housekeeping gene GAPDH are presented. The sequences of primers used are MICA: F: 5′-ACTTGACAGGGAACGGAAAG-3′, R: 5′-GAGGAAGAGCTCCCCATCGT-3′; MICB: F: 5′-CCGAGGACTTGACAGAGAAT-3′, R: 5′-CTGCTGTCTTCATGGATCTC-3′; GAPDH: F: 5′- GTGAACCATGAGAAGTATGACAAC-3′, R: 5′- CATGAGTCCTTCCACGATACC-3′.

### 4.7. Statistical Analysis

Statistical analysis was conducted using GraphPad Prism 8 software. Comparisons between groups were performed using the nonparametric Mann–Whitney test and the nonparametric Wilcoxon signed-rank test. Differences were considered statistically significant at *p* < 0.05.

## 5. Conclusions

JEG-3 choriocarcinoma cells and NK-92 cells express MICs. NK cells also highly express the NKG2D receptor. TSA-treated JEG-3 cells show decreased MIC expression, accompanied by the release of sMICB. Nevertheless, the reduction in MIC expression by TSA-treated JEG-3 cells did not protect them from the cytotoxic activity of NK cells. A decrease in the susceptibility of TSA-treated JEG-3 cells to NK cell-mediated cytotoxicity was observed only when NK cells were activated with IL-12. Thus, IL-12-activated NK cells lose the ability to efficiently kill TSA-treated choriocarcinoma cells through a mechanism involving MICs.

## Figures and Tables

**Table 1:** Antibodies used in this study.

Marker	Fluorochrome	Cat. No	Figure	Supplementary Figure
CD45	BV480	BD, Franklin Lakes, NJ, USA, 566115	—	—
NKG2D	APC	BD, Franklin Lakes, NJ, USA, 558071	Figure 2d,e	Figure S3(a1,a2)
NKG2D	APC	Thermo Fisher, Waltham, Ma, USA 17-5878-81	Figure 2f,g	Figure S3(d1,d2)
MICA	AF700	R&D, Minneapolis, MN, USA, FAB1300N	Figure 1a,b and Figure 2a,b	Figures S2(a1,a2) and S3(b1,b2)
MICA	RB705	BD, Franklin Lakes, NJ, USA, 757415	Figure 2c	Figures S2(c1,c2) and S3(e1,e2)
MICB	AF750	R&D, Minneapolis, MN, USA, FAB1599S	Figure 1c,d and Figure 2h	Figures S2(b1,b2) and S3(c1,c2)
MICB	AF488	R&D, Minneapolis, MN, USA, FAB1599G	Figure 1e and Figure 2i,j	Figures S2(d1,d2) and S3(f1,f2)

## Data Availability

The data presented in this study are available on request from the corresponding author.

## Supplementary Materials

The following supporting information can be downloaded at https://www.mdpi.com/article/10.3390/ijms26178400/s1.

## Abbreviations

The following abbreviations are used in this manuscript:

NK cells	natural killer cells
MICs	MICA/B proteins
TSA	trichostatin A
sMICs	soluble MICA/B proteins
HDACi	histone deacetylase inhibitor
rMICA/B	recombinant MICA/B proteins
Anti-MICA/B	antibodies to MICA/B proteins
PBMCs	peripheral blood mononuclear cells

## References

[1] Venkataraman G.M., Suciu D., Groh V., Boss J.M., Spies T. (2007). Promoter region architecture and transcriptional regulation of the genes for the MHC class I-related chain A and B ligands of NKG2D. J. Immunol..

[2] Zhao Y., Chen N., Yu Y., Zhou L., Niu C., Liu Y., Tian H., Lv Z., Han F., Cui J. (2017). Prognostic value of MICA/B in cancers: A systematic review and meta-analysis. Oncotarget.

[3] Torn C., Gupta M., Sanjeevi C.B., Aberg A., Frid A., Landin-Olsson M. (2004). Different HLA-DR-DQ and MHC class I chain-related gene A (MICA) genotypes in autoimmune and nonautoimmune gestational diabetes in a Swedish population. Hum. Immunol..

[4] Vinnars M.T., Bjork E., Nagaev I., Ottander U., Bremme K., Holmlund U., Sverremark-Ekstrom E., Mincheva-Nilsson L. (2018). Enhanced Th1 and inflammatory mRNA responses upregulate NK cell cytotoxicity and NKG2D ligand expression in human pre-eclamptic placenta and target it for NK cell attack. Am. J. Reprod. Immunol..

[5] Apps R., Gardner L., Traherne J., Male V., Moffett A. (2008). Natural-killer cell ligands at the maternal-fetal interface: UL-16 binding proteins, MHC class-I chain related molecules, HLA-F and CD48. Hum. Reprod..

[6] Bauer S., Groh V., Wu J., Steinle A., Phillips J.H., Lanier L.L., Spies T. (1999). Activation of NK cells and T cells by NKG2D, a receptor for stress-inducible MICA. Science.

[7] Ogasawara K., Lanier L.L. (2005). NKG2D in NK and T cell-mediated immunity. J. Clin. Immunol..

[8] Dhar P., Wu J.D. (2018). NKG2D and its ligands in cancer. Curr. Opin. Immunol..

[9] Sharma N., Trinidad C.V., Trembath A.P., Markiewicz M.A. (2017). NKG2D Signaling between Human NK Cells Enhances TACE-Mediated TNF-alpha Release. J. Immunol..

[10] Li P., Morris D.L., Willcox B.E., Steinle A., Spies T., Strong R.K. (2001). Complex structure of the activating immunoreceptor NKG2D and its MHC class I-like ligand MICA. Nat. Immunol..

[11] Graham D.B., Cella M., Giurisato E., Fujikawa K., Miletic A.V., Kloeppel T., Brim K., Takai T., Shaw A.S., Colonna M. (2006). Vav1 controls DAP10-mediated natural cytotoxicity by regulating actin and microtubule dynamics. J. Immunol..

[12] Watzl C., Urlaub D. (2012). Molecular mechanisms of natural killer cell regulation. Front. Biosci..

[13] Hedlund M., Stenqvist A.C., Nagaeva O., Kjellberg L., Wulff M., Baranov V., Mincheva-Nilsson L. (2009). Human placenta expresses and secretes NKG2D ligands via exosomes that down-modulate the cognate receptor expression: Evidence for immunosuppressive function. J. Immunol..

[14] Ashiru O., Boutet P., Fernandez-Messina L., Aguera-Gonzalez S., Skepper J.N., Vales-Gomez M., Reyburn H.T. (2010). Natural killer cell cytotoxicity is suppressed by exposure to the human NKG2D ligand MICA*008 that is shed by tumor cells in exosomes. Cancer Res..

[15] Boutet P., Aguera-Gonzalez S., Atkinson S., Pennington C.J., Edwards D.R., Murphy G., Reyburn H.T., Vales-Gomez M. (2009). Cutting edge: The metalloproteinase ADAM17/TNF-alpha-converting enzyme regulates proteolytic shedding of the MHC class I-related chain B protein. J. Immunol..

[16] Yang Y., Wang Y., Zeng X., Ma X.J., Zhao Y., Qiao J., Cao B., Li Y.X., Ji L., Wang Y.L. (2012). Self-control of HGF regulation on human trophoblast cell invasion via enhancing c-Met receptor shedding by ADAM10 and ADAM17. J. Clin. Endocrinol. Metab..

[17] Groh V., Wu J., Yee C., Spies T. (2002). Tumour-derived soluble MIC ligands impair expression of NKG2D and T-cell activation. Nature.

[18] Ferrari de Andrade L., Tay R.E., Pan D., Luoma A.M., Ito Y., Badrinath S., Tsoucas D., Franz B., May K.F., Harvey C.J. (2018). Antibody-mediated inhibition of MICA and MICB shedding promotes NK cell-driven tumor immunity. Science.

[19] Kato N., Tanaka J., Sugita J., Toubai T., Miura Y., Ibata M., Syono Y., Ota S., Kondo T., Asaka M. (2007). Regulation of the expression of MHC class I-related chain A, B (MICA, MICB) via chromatin remodeling and its impact on the susceptibility of leukemic cells to the cytotoxicity of NKG2D-expressing cells. Leukemia.

[20] Lin D., Lavender H., Soilleux E.J., O’Callaghan C.A. (2012). NF-kappaB regulates MICA gene transcription in endothelial cell through a genetically inhibitable control site. J. Biol. Chem..

[21] Langhans B., Ahrendt M., Nattermann J., Sauerbruch T., Spengler U. (2005). Comparative study of NK cell-mediated cytotoxicity using radioactive and flow cytometric cytotoxicity assays. J. Immunol. Methods.

[22] Jurisic V., Spuzic I., Konjevic G. (1999). A comparison of the NK cell cytotoxicity with effects of TNF-alpha against K-562 cells, determined by LDH release assay. Cancer Lett..

[23] Drvar V., Curko-Cofek B., Karleusa L., Aralica M., Rogoznica M., Kehler T., Legovic D., Rukavina D., Laskarin G. (2022). Granulysin expression and granulysin-mediated apoptosis in the peripheral blood of osteoarthritis patients. Biomed. Rep..

[24] Huang S.Y., Chiang C.H., Chen F.P., Yu C.L. (2011). The alteration of placental-derived soluble MHC class I chain-related protein A and B during pregnancy. Acta Obstet. Gynecol. Scand..

[25] Marlin R., Duriez M., Berkane N., de Truchis C., Madec Y., Rey-Cuille M.A., Cummings J.S., Cannou C., Quillay H., Barre-Sinoussi F. (2012). Dynamic shift from CD85j/ILT-2 to NKG2D NK receptor expression pattern on human decidual NK during the first trimester of pregnancy. PLoS ONE.

[26] Molinero L.L., Domaica C.I., Fuertes M.B., Girart M.V., Rossi L.E., Zwirner N.W. (2006). Intracellular expression of MICA in activated CD4 T lymphocytes and protection from NK cell-mediated MICA-dependent cytotoxicity. Hum. Immunol..

[27] Molinero L.L., Fuertes M.B., Rabinovich G.A., Fainboim L., Zwirner N.W. (2002). Activation-induced expression of MICA on T lymphocytes involves engagement of CD3 and CD28. J. Leukoc. Biol..

[28] Molinero L.L., Fuertes M.B., Girart M.V., Fainboim L., Rabinovich G.A., Costas M.A., Zwirner N.W. (2004). NF-kappa B regulates expression of the MHC class I-related chain A gene in activated T lymphocytes. J. Immunol..

[29] Stolbovaya A.Y., Pinevich A.A., Gryazeva I.V., Krutetskaya I.Y., Zharinov G.M., Morozova M.A., Kneev A.Y., Terekhina L.A., Ishchuk S.A., Shashkova O.A. (2025). Detection and Quantification of Polymorphic MICA and MICB Molecules in Immunoassays: Initial Insights. HLA.

[30] Klumkrathok K., Jumnainsong A., Leelayuwat C. (2013). Allelic MHC class I chain related B (MICB) molecules affect the binding to the human cytomegalovirus (HCMV) unique long 16 (UL16) protein: Implications for immune surveillance. J. Microbiol..

[31] Carapito R., Bahram S. (2015). Genetics, genomics, and evolutionary biology of NKG2D ligands. Immunol. Rev..

[32] Du C., Bevers J., Cook R., Lombana T.N., Rajasekaran K., Matsumoto M., Spiess C., Kim J.M., Ye Z. (2019). MICA immune complex formed with alpha 3 domain-specific antibody activates human NK cells in a Fc-dependent manner. J. Immunother. Cancer.

[33] Gong J.H., Maki G., Klingemann H.G. (1994). Characterization of a human cell line (NK-92) with phenotypical and functional characteristics of activated natural killer cells. Leukemia.

[34] Wu J.D., Higgins L.M., Steinle A., Cosman D., Haugk K., Plymate S.R. (2004). Prevalent expression of the immunostimulatory MHC class I chain-related molecule is counteracted by shedding in prostate cancer. J. Clin. Investig..

[35] Dhar P., Basher F., Ji Z., Huang L., Qin S., Wainwright D.A., Robinson J., Hagler S., Zhou J., MacKay S. (2021). Tumor-derived NKG2D ligand sMIC reprograms NK cells to an inflammatory phenotype through CBM signalosome activation. Commun. Biol..

[36] Hakam M.S., Miranda-Sayago J.M., Hayrabedyan S., Todorova K., Spencer P.S., Jabeen A., Barnea E.R., Fernandez N. (2017). Preimplantation Factor (PIF) Promotes HLA-G, -E, -F, -C Expression in JEG-3 Choriocarcinoma Cells and Endogenous Progesterone Activity. Cell Physiol. Biochem..

[37] Vacca P., Cantoni C., Prato C., Fulcheri E., Moretta A., Moretta L., Mingari M.C. (2008). Regulatory role of NKp44, NKp46, DNAM-1 and NKG2D receptors in the interaction between NK cells and trophoblast cells. Evidence for divergent functional profiles of decidual versus peripheral NK cells. Int. Immunol..

[38] Zhang C., Zhang J., Niu J., Zhou Z., Zhang J., Tian Z. (2008). Interleukin-12 improves cytotoxicity of natural killer cells via upregulated expression of NKG2D. Hum. Immunol..

[39] Kohler P.O., Bridson W.E. (1971). Isolation of hormone-producing clonal lines of human choriocarcinoma. J. Clin. Endocrinol. Metab..

[40] Lozzio B.B., Lozzio C.B. (1979). Properties and usefulness of the original K-562 human myelogenous leukemia cell line. Leuk. Res..

[41] Shin S., Kim M., Lee S.J., Park K.S., Lee C.H. (2017). Trichostatin A Sensitizes Hepatocellular Carcinoma Cells to Enhanced NK Cell-mediated Killing by Regulating Immune-related Genes. Cancer Genom. Proteom..

[42] Flieger D., Gruber R., Schlimok G., Reiter C., Pantel K., Riethmuller G. (1995). A novel non-radioactive cellular cytotoxicity test based on the differential assessment of living and killed target and effector cells. J. Immunol. Methods.

