# Supplementary material for: The Role of MICA/B Molecules and the NKG2D Receptor in the Interaction Between NK-92 Cells and JEG-3 Cells
Source: Int J Mol Sci. 2025 Aug 29;26(17):8400. doi: 10.3390/ijms26178400 (PMC12428272; doi:10.3390/ijms26178400)
Supplement: Supplementary file 1 [file ijms-26-08400-s001.zip › ijms-3778405-supplementary.pdf]

International Journal of
Molecular Sciences

MDPI

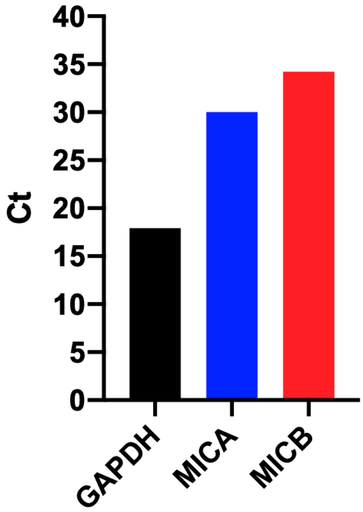

Figure S1. Cycle threshold (Ct) values for *GAPDH*, *MICA*, and *MICB* in JEG-3 cells measured by qPCR. Samples with Ct values below 35 were considered positive for gene expression.

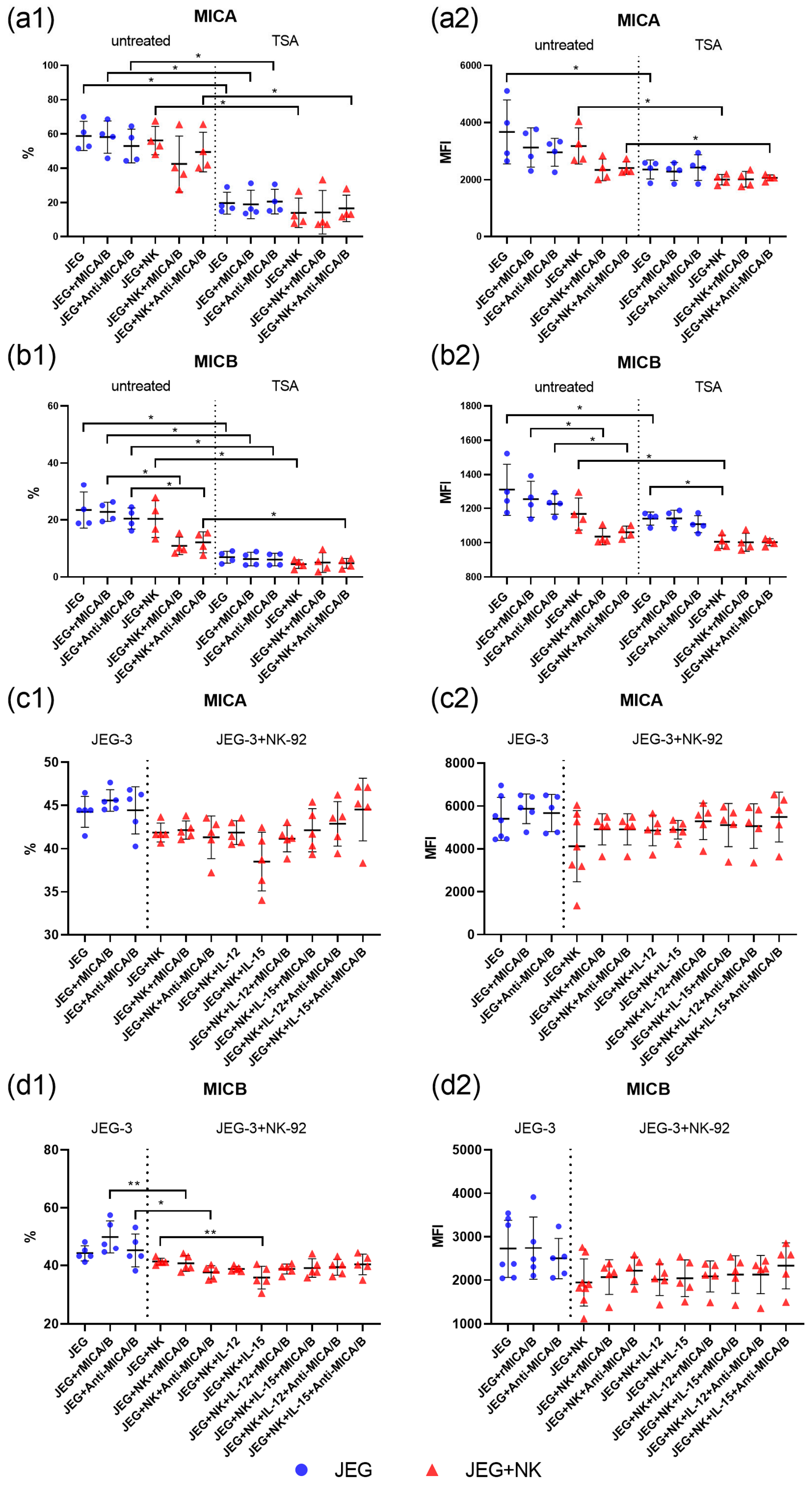

Figure S2. Phenotype of JEG-3 cells under mono- and co-culture conditions. The figure shows the percentage (%) of JEG-3 cells expressing MICA (a1, c1) and MICB (b1, d1) proteins, and the intensity of expression (MFI) of MICA (a2, c2) and MICB (b2, d2). Two independent series of experiments

were conducted. In the first, the phenotype of TSA-treated JEG-3 and NK-92 cells was analyzed (a1, a2, b1, b2). In the second, changes after co-culturing JEG-3 cells with IL12/IL-15-activated NK-92 cells were measured (c1, c2, d1, d2). MFI – Median Fluorescence Intensity; untreated – TSA-untreated cells; TSA – TSA-treated cells; JEG – JEG-3 cells, NK – NK-92 cells. Significant differences: * – $p < 0.05$; ** – < 0.01. There were four biological replicates with one technical replicate in each experiment.

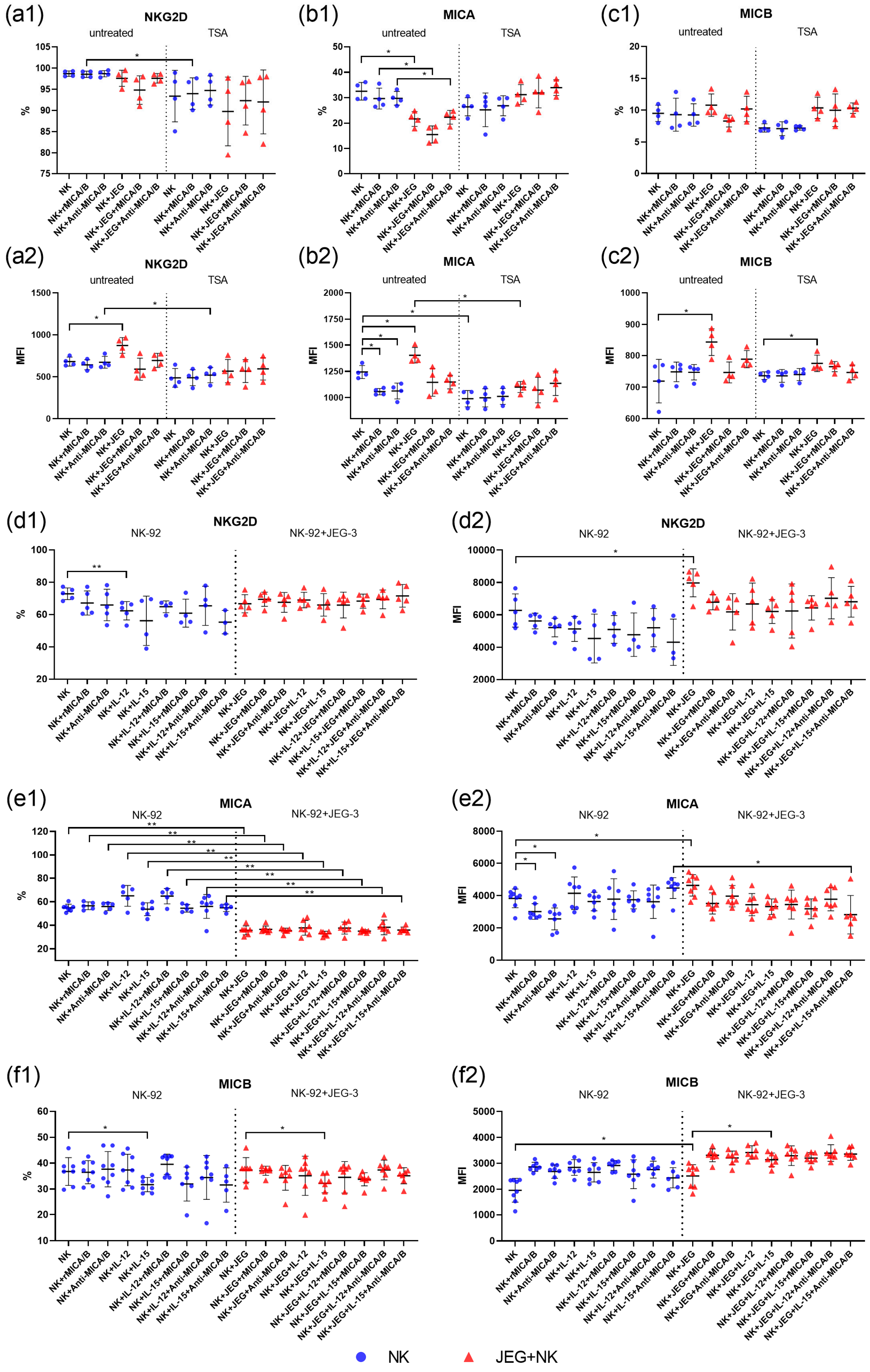
(a1)
NKG2D
untreated
TSA
%
NK
NK+rMICA/B
NK+Anti-MICA/B
NK+JEG
NK+JEG+rMICA/B
NK+JEG+Anti-MICA/B
(b1)
MICA
(c1)
MICB
(a2)
NKG2D
MFI
(b2)
MICA
(c2)
MICB
(d1)
NKG2D
NK-92
NK-92+JEG-3
NK+IL-12
NK+IL-15
NK+IL-12+rMICA/B
NK+IL-15+rMICA/B
NK+IL-12+Anti-MICA/B
NK+IL-15+Anti-MICA/B
NK+JEG+IL-12
NK+JEG+IL-15
NK+JEG+IL-12+rMICA/B
NK+JEG+IL-15+rMICA/B
NK+JEG+IL-12+Anti-MICA/B
NK+JEG+IL-15+Anti-MICA/B
(d2)
NKG2D
(e1)
MICA
(e2)
MICA
(f1)
MICB
(f2)
MICB
NK
JEG+NK

Figure S3. Phenotype of NK-92 cells under mono- and co-culture conditions. The figure shows the percentage (%) of NK-92 cells expressing NKG2D (a1, d1), MICA (b1, e1), and MICB (c1, f1) proteins, and the intensity of expression (MFI) of NKG2D (a2, d2), MICA (b2, e2), and MICB (c2, f2). Two independent series of experiments were conducted. In the first, the phenotype of TSA-treated JEG-3 and NK-92 cells was analyzed (a1, a2, b1, b2, c1, c2). In the second, changes after co-culturing JEG-3 cells with IL12/IL-15-activated NK-92 cells were measured (d1, d2, e1, e2, f1, f2). MFI – Median Fluorescence Intensity; untreated – TSA-untreated cells; TSA – TSA-treated cells; JEG – JEG-3 cells, NK – NK-92 cells. Significant differences: * – $p < 0.05$; ** – < 0.01. There were four biological replicates with one technical replicate in each experiment.

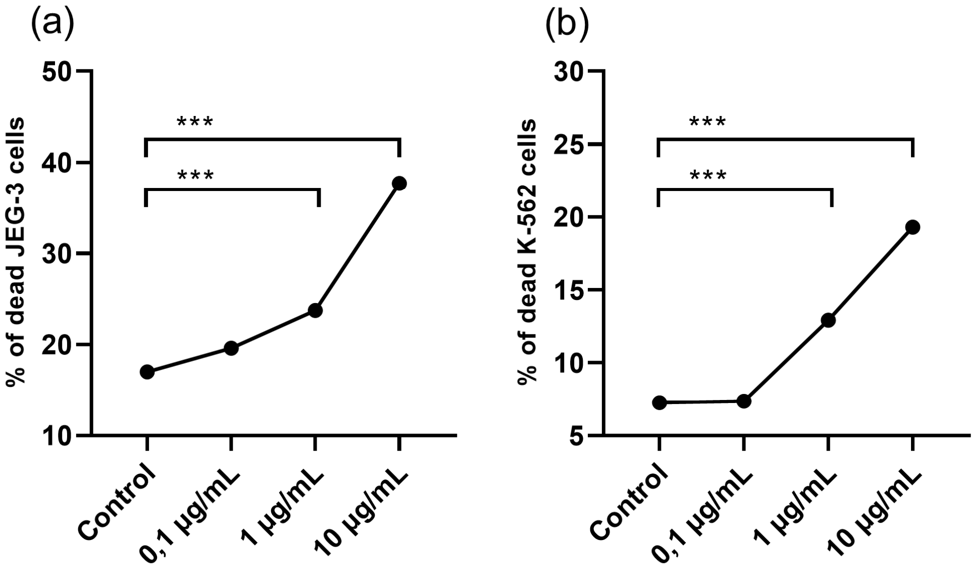

Figure S4. The percentage of dead JEG-3 (a) and K-562 (b) cells in the presence of various concentrations of TSA. Significant differences: *** – $p < 0.001$.

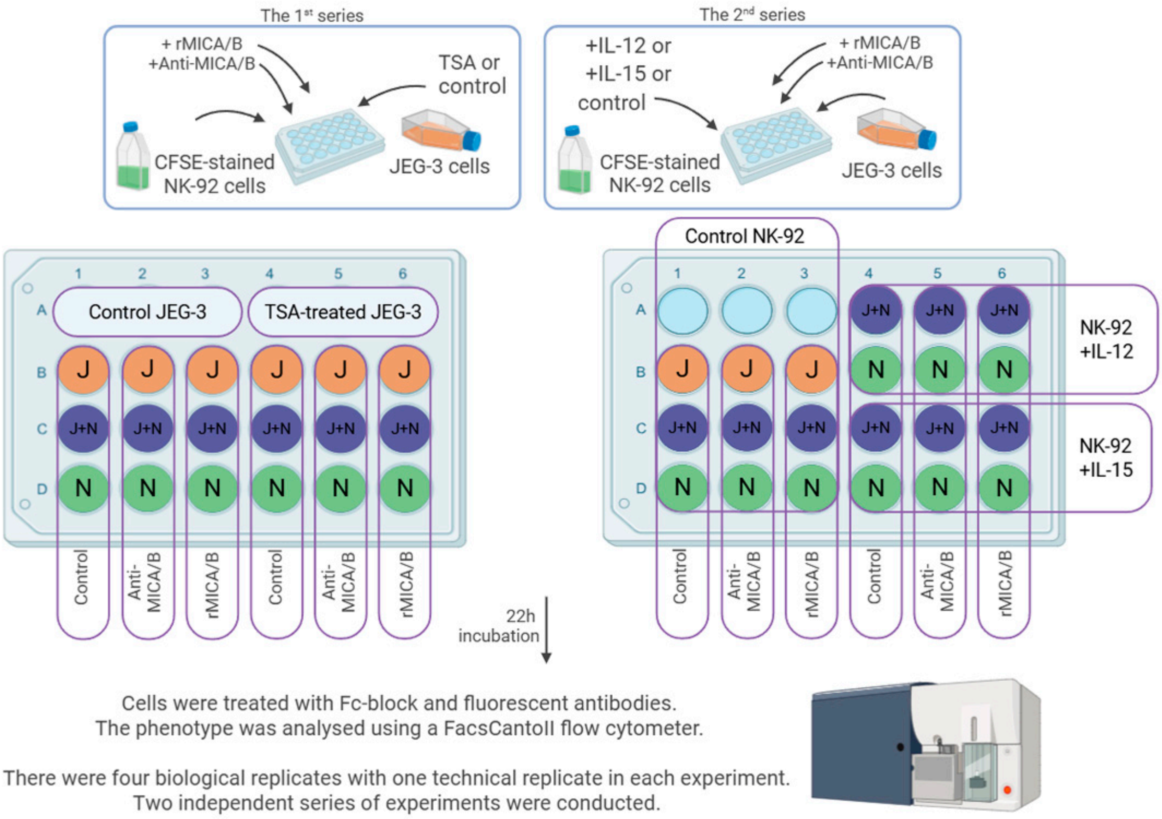

Figure S5. Scheme describing the procedure for assessing cell phenotype. Two independent series of experiments were conducted. In the first, the phenotype of TSA-treated JEG-3 and NK-92 cells was analyzed. In the second, changes after co-culturing JEG-3 cells with IL12/IL-15-activated NK-92 cells were measured. Before the experiment, NK-92 cells in the second series were treated with IL-12 or IL-15 for 22 hours. In both series, JEG-3 cells were seeded into 24-well plates. On the day of the experiment, NK-92 cells were stained with CFSE solution. Subsequently, rMICA/B or Anti-MICA/B were added to some wells with JEG-3 cells, as shown in the plate schemes. After 20 minutes, NK-92 cells were added to the designated wells (N and J+N): untreated cells in the first series, and untreated (control) or activated with IL-12 or IL-15 in the second series. Additionally, in the first series, TSA was added to all wells indicated on the plate scheme. Following 22 hours of incubation, the cells were harvested from the plates, treated with an Fc-block solution, and stained with fluorescent monoclonal antibodies. The phenotype was analyzed using a FACSCanto II flow cytometer. The image is created in BioRender.

International Journal of *Molecular Sciences*

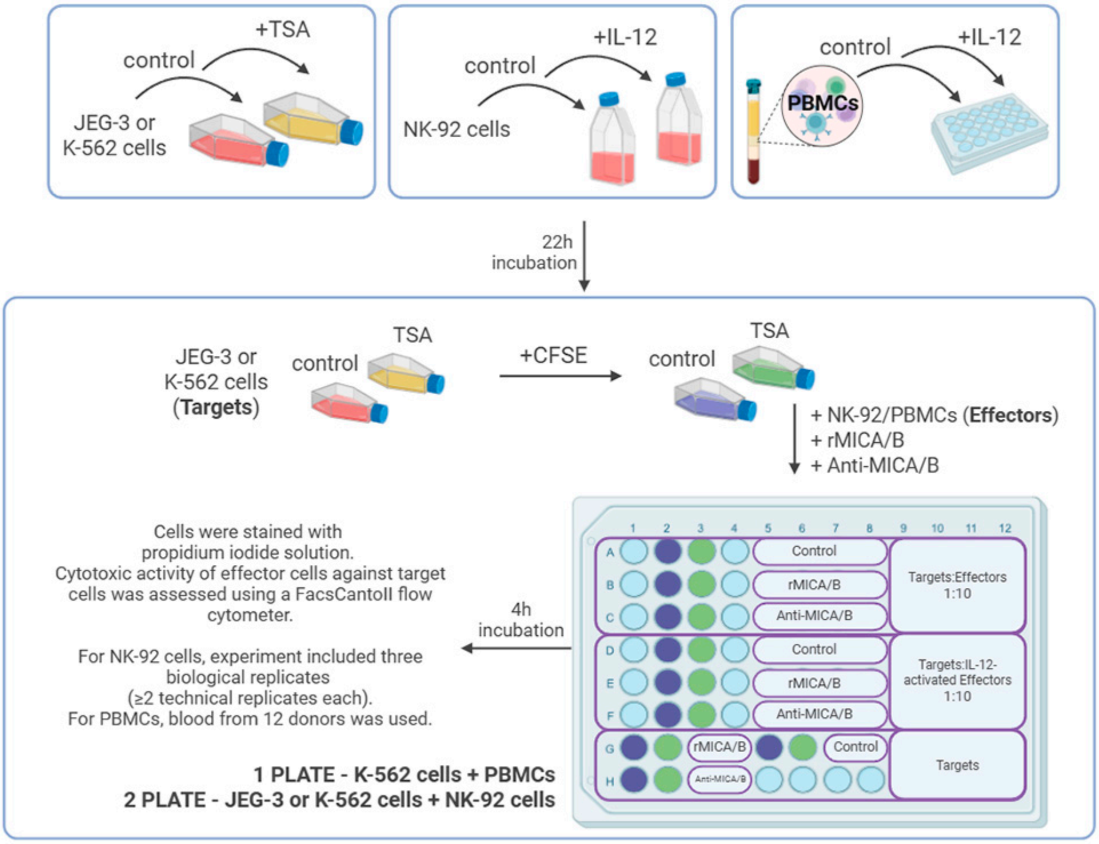

Figure S6. Scheme describing the procedure for assessing the cytotoxic activity of effector cells (NK-92 cells/PBMCs) against target cells (JEG-3/K-562 cells). Two independent series of experiments were conducted. In the first series, the cytotoxicity of PBMCs to K-562 cells was assessed. In the second, the cytotoxicity of NK-92 cells to JEG-3 or K-562 cells was evaluated. In both series, target cells were seeded into flasks prior to the experiment; TSA was added to some of them. NK-92 cells were seeded into flasks, while PBMCs were seeded into 24-well plates; IL-12 was added to activate some cells. After 22 hours of incubation, target cells were stained with CFSE solution and subsequently seeded into 96-well plates. rMICA/B or Anti-MICA/B were then added to some wells, as shown in the plate schemes. After 20 minutes, control and IL-12-activated effector cells were added to the designated wells at an effector-to-target ratio of 10:1. The plate was centrifuged for 5 minutes at 100 × g to facilitate cell-cell contact. After 4 hours of incubation, the cells were stained with propidium iodide solution. The relative number of dead target cells was assessed using a FACSCantoII flow cytometer. The image is created in BioRender.

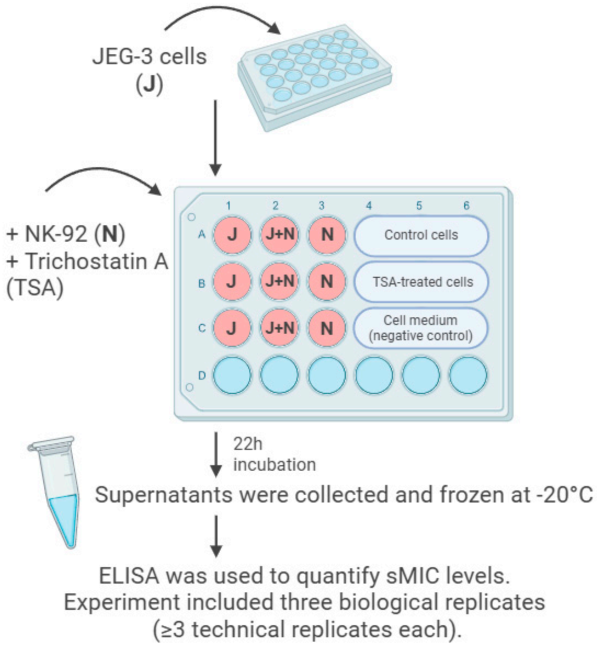

Figure S7. Scheme describing the procedure for assessing the soluble form of MIC (sMIC) in cell supernatants. JEG-3 cells were seeded into 24-well plate and incubated for 22 hours. After that, NK-92 cells were added to the designated wells (N and J+N). TSA was added to the wells in row B. Additionally, culture medium was added to a separate row of wells and used as a negative control. After 22 hours of incubation, the supernatants were collected and frozen at -20°C. The soluble form of MIC in the samples was quantified using a two-site ELISA.
